# Multidisciplinary treatment of advanced or recurrent solid pseudopapillary neoplasm of the pancreas: three case reports

**DOI:** 10.1186/s40792-022-01358-0

**Published:** 2022-01-10

**Authors:** Kiyonori Tanoue, Yuko Mataki, Hiroshi Kurahara, Tetsuya Idichi, Yota Kawasaki, Yoichi Yamasaki, Yoshiaki Kita, Yuto Hozaka, Hideyuki Oi, Akihiro Nakajo, Takaaki Arigami, Kosei Maemura, Takao Ohtsuka

**Affiliations:** 1 Department of Digestive Surgery, Breast and Thyroid Surgery, Kagoshima University Graduate School of Medical and Dental Sciences, 8-35-1 Sakuragaoka, Kagoshima, 890-8520 Japan; 2 Department of Onco-Biological Surgery, Kagoshima University Graduate School of Medical and Dental Sciences, Kagoshima, Japan; 3 Department of Digestive Surgery, Kagoshima City Hospital, Kagoshima, Japan

**Keywords:** Solid pseudopapillary neoplasm, Frantz tumor, Malignancy, Multidisciplinary treatment

## Abstract

**Background:**

Solid pseudopapillary neoplasm (SPN) is a rare pancreatic tumor that predominantly affects young females. Prognosis is excellent; however, 10–15% of patients show metastasis at the time of surgery or develop tumor recurrence after pancreatectomy.

**Case presentation:**

We reviewed the clinical course of three patients with advanced or recurrent SPN and subsequently underwent multidisciplinary treatment at our institution between 2002 and 2019. The primary tumor was resected in all three patients, and metastases were also resected if indicated. Intensive combined therapy, including re-resection, chemotherapy, ablation, arterial chemoembolization, and radiation therapy, allowed all patients to survive for a long time. The literature review showed that resection seems to be more effective than other treatments for metastatic SPN.

**Conclusions:**

Multidisciplinary treatment, including resection, may improve the prognosis of patients with SPN with recurrence or metastasis.

## Background

Solid pseudopapillary neoplasm (SPN) of the pancreas, also known as Frantz’s tumor, is rare and usually carries a favorable prognosis, with a 5-year survival rate of up to 97% [1–3]. However, up to 10–15% of affected patients have distant metastasis [4]. Because of its rarity, many controversies remain regarding treatment for recurrent or metastatic SPN. Herein, we report three patients with recurrent and metastatic SPN who underwent multidisciplinary treatment and discuss the efficacy of the treatment using a literature review.

## Case presentation

**Fig. 1:**
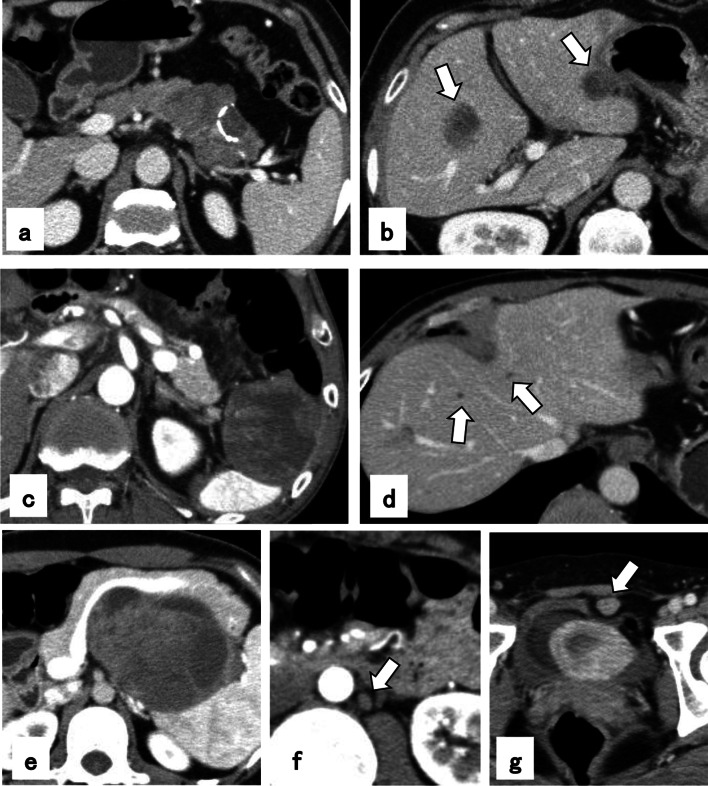
**a** Computed tomography (CT) image of the primary tumor in a solid pseudopapillary neoplasm (SPN) (Patient No. 1). **b** CT image of liver metastases 10 months after resection of the primary tumor (Patient No. 1). **c** CT image of the main tumor in SPN (Patient No. 2). **d** CT image of multiple liver metastases 9 months after resection of the primary tumor (Patient No. 2). **e** CT image of the primary tumor in SPN (Patient No. 3). **f** CT image of synchronous para-aortic lymph node metastasis (Patient No. 3). **g** CT image of peritoneal wall metastasis in the anterior wall of the urinary bladder (Patient No. 3)

Patient No. 1 was a 49-year-old female who underwent computed tomography (CT) scan for a pancreatic tumor detected by abdominal ultrasound during her annual check-up. The CT scan highlighted a pancreatic tumor in the pancreatic tail, which was 7 cm in diameter, and multiple liver metastases in subsegments 2, 4, and 7 (Fig. 1a). After chemotherapy using gemcitabine (GEM) plus S-1, which led to a slight increase in the size of the tumor while no other new lesion was formed, we performed distal pancreatectomy (DP) and resection of all the metastatic liver tumors. Pathological examination showed SPN with a 3% Ki-67 index and liver metastases of SPN. Ten months later, solitary hepatic recurrences were observed in subsegments 2 and 4 (Fig. 1b). The metastasis in subsegment 2 was resected, and that in subsegment 4 was treated by radiofrequency ablation (RFA). Sixteen months later, re-recurrence was noted in multiple sites of the liver, which was a contraindication for surgery, and she underwent transcatheter arterial chemoembolization (TACE) as well as transcatheter arterial infusion (TAI) of epirubicin hydrochloride 9 times in 34 months. Thereafter, she underwent whole-liver irradiation, and she has survived for an additional 11 months to date (a total of 111 months after the initial diagnosis).

Patient No. 2 was a 57-year-old male who underwent a CT scan for gastric ulcer perforation, and a pancreatic tumor measuring 7 cm was incidentally detected (Fig. 1c). After emergency surgery for the perforated gastric ulcer, the patient underwent DP, and the pathological results revealed SPN with capsule invasion. Nine months later, the patient suffered from multiple liver metastases in the subsegments 4, 6, 7, and 8, with a total number of 9 metastases (Fig. 1d). After 2 months of chemotherapy using GEM plus S-1, which led to a slight increase in the size of the metastases, we performed multiple partial resections of the liver to achieve R0 resection. This patient had survived for 50 months without any sign of re-recurrence to date (a total of 79 months after the initial diagnosis).

Patient No. 3 was a 30-year-old female who underwent a CT scan for suspected tuberculosis. The CT scan revealed a pancreatic tumor, measuring 10 cm in diameter with para-aortic lymph node (#16b1 latero) and abdominal wall metastases (Fig. 1e–g). We resected the pancreatic tumor, together with a sampling of the metastatic lymph nodes and the abdominal wall. Pathological results revealed SPN with a < 1% Ki-67 index and capsule invasion. Then, we performed a histoculture drug response assay (HDRA), a chemosensitivity test reported by Hoffman [5]. The test showed high sensitivity to paclitaxel, and thus, the patient received chemotherapy using S-1 plus paclitaxel. Paclitaxel treatment for SPN is not covered by the Japanese National Health Insurance, so this patient provided informed consent and paid for the treatment. Thereafter, the lymph node metastases showed stability for 163 months. Because of the increasing accumulation in the para-aortic lymph node (#16b1 latero) on fluorodeoxyglucose position emission tomography without any other lesion, we performed resection of the metastatic lymph nodes, and thereafter, she has survived for an additional 14 months without any sign of recurrence to date (total 183 months after initial diagnosis).

## Discussion

Because metastasis or recurrence of SPN is rare, there has been little investigation into its treatment. Resection is also considered optimal for recurrent and metastatic lesions but has little supportive evidence. According to the data from 77 SPN patients who received treatment for distant metastasis, recurrence, and/or invasions of adjacent organs in previous reports [4, 6–35] (Table 1), patients who underwent resection (*n* = 57) had a significantly better prognosis than patients who underwent non-surgical treatment (*n* = 20) (median overall survival, 36 months vs. 31 months, *p* = 0.0244). Although selection bias may exist in the literature review, resection of metastatic or recurrent SPN, if applicable, may contribute to improving the patient’s prognosis. Among the above-mentioned 77 patients, 33 were treated with resection only, while 44 patients were treated with non-surgical treatment. In those 44 patients, the efficacy of non-surgical treatment for SPN was evaluated for each treatment modality (Table 1).

**Table 1:** Characteristics of 77 SPN patients with malignant behavior (distant metastasis, recurrence and/or invasion) in the literature

Patient characteristics	Number of patients	References
Age, median years (range)	24 (7–66)*	[4, 6–35]
Male/female/unknown	8 / 54 / 15	[4, 6–35]
Location of main tumor, head/body or tail/unknown	13 / 36 / 28	[4, 6–35]
Metastatic and recurrent site (%)		
Liver	44 (57.1)	[4, 6–35]
Lung	3 (3.9)	[4, 6–35]
Peritoneum	19 (24.7)	[4, 6–35]
Lymph node	9 (11.7)	[4, 6–35]
Local	18 (23.4)	[4, 6–35]
Others	6 (7.8)	[4, 6–35]
Non-surgical treatment effect, tumor reduction/no response/missing data (total number)		
GEM-based regimen	0 / 6 / 4 (10)	[10, 17, 19, 21, 23, 29]
CDDP-based regimen	2 / 2 / 2 (6)	[10, 17, 18]
5-FU-based regimen	0 / 3 / 2 (5)	[8, 10, 20]
CDDP + 5-FU	2 / 0 / 3 (5)	[10, 25, 26]
Interferon	0 / 1 / 2 (3)	[10, 32]
Tamoxifen	0 / 2 / 0 (2)	[8, 10]
GEM + CDDP	1 / 1 / 0 (2)	[10, 31]
Somatostatin	0 / 1 / 0 (1)	[10]
Capecitabine	0 / 1 / 0 (1)	[10]
Paclitaxel	0 / 1 / 0 (1)	[19]
Thalidomide	0 / 1 / 0 (1)	[10]
Imatinib	0 / 1 / 0 (1)	[10]
Oxaliplatin-based regimen	0 / 1 / 0 (1)	[10]
Erlotinib	0 / 1 / 0 (1)	[23]
RFA	0 / 3 / 4 (7)	[7, 10, 17]
RT	5 / 0 / 0 (5)	[22, 32–35]
CRT	0 / 1 / 1 (2)	[10]
TAI	0 / 1 / 1 (2)	[10, 23]
TACE	1 / 0 / 1 (2)	[8, 23]
HIPEC	0 / 1 / 1 (2)	[11, 30]
TAE	0 / 1 / 0 (1)	[10]
MCN	0 / 1 / 0 (1)	[10]
SIRT	0 / 0 / 1 (1)	[17]
CS-PHP	1 / 0 / 0 (1)	[31]
Proton beam therapy	1 / 0 / 0 (1)	[29]

*GEM* gemcitabine, *CDDP* cisplatin, *5-FU* 5-fluorouracil, *RFA* radiofrequency ablation, *RT* radiation therapy, *CRT* chemoradiotherapy, *TAI* transcatheter arterial infusion, *TACE* transcatheter arterial chemoembolization, *HIPEC* hyperthermic intraperitoneal chemotherapy, *TAE* transcatheter arterial embolization, *MCN* microwave coagulo-necrotic therapy, *SIRT* selective internal radiation therapy, *CS-PHP* chemosaturation with percutaneous hepatic perfusion

^*^Only 62 patients' data were available

Soloni et al. reported that cisplatin, 5-fluorouracil (5-FU), and GEM were the most frequently used chemotherapeutic agents in treating unresectable SPN [10]. In their review, some benefit was observed following cisplatin administration in 5 of 8 patients, 5-FU in 3 of 8 patients, and GEM in 2 of 6 patients. However, SPN is a slow-growing tumor and usually shows stable condition even without chemotherapeutic agents; therefore, the efficacy of these drugs in SPN remains unclear. We used GEM plus S-1 in patients No. 1 and 2, according to the regimen for pancreatic ductal adenocarcinoma; however, the tumor had increased slightly in size after chemotherapy in both patients. It is anticipated that there will be many patients with SPN who will not respond to these chemotherapies. In fact, our data from the literature indicate that only a few patients have achieved tumor reduction with chemotherapy and that there is no clearly effective regimen for SPN. However, it is possible that cisplatin may be effective, as all regimens that showed tumor reduction included cisplatin (Table 1).

In this report, one patient showed sensitivity to paclitaxel in HDRA, and para-aortic lymph node metastasis kept the stable disease in the long-term after paclitaxel treatment. Only one previous study [21] reported chemosensitivity tests for recurrent and metastatic SPN. In that study, the collagen gel droplet embedded culture drug sensitivity test showed that GEM was the most sensitive agent, and they subsequently used it as adjuvant chemotherapy after resection of recurrent lesions. Their patient had no recurrence for 18 months after the second operation. In the future, such patient-specific chemosensitivity tests may be useful for the adequate selection of chemotherapeutic agents in treating malignant SPN.

The efficacy of other non-surgical treatments such as TACE, TAI, and RFA was not definitive in our patients, nor was it evident in data from the literature. However, there are 5 case reports in the literature about the reduction effect of radiation therapy on SPN [22, 32–35], and Kodama et al. [29] reported that in a patient who had liver and lymph node metastases 15 years after an initial operation, proton therapy reduced his tumor size despite the resistance to GEM plus nab-paclitaxel (Table 1). Liver transplantation was also reported to be useful for treating multiple liver metastases from SPN in four cases [16, 22, 23, 36]. These treatments could be an option for unresectable SPN.

## Conclusion

Multidisciplinary treatment, including resection, may improve the prognosis of patients with SPN with recurrence or metastasis.

## Data Availability

The datasets supporting the conclusions of this article are included within the article and its additional files.

## Abbreviations

5-FU: 5-Fluorouracil
CT: Computed tomography
DP: Distal pancreatectomy
GEM: Gemcitabine
HDRA: Histoculture drug response assay
RFA: Radiofrequency ablation
SPN: Solid pseudopapillary neoplasm
TACE: Transcatheter arterial chemoembolization
TAI: Transcatheter arterial infusion
